# Combined effects of *FH* (E404D) and *ACOX2* (R409H) cause metabolic defects in primary cardiac malignant tumor

**DOI:** 10.1038/s41420-018-0072-3

**Published:** 2018-07-23

**Authors:** Xiangyu Zhou, Mengjia Xu, Weijia Zeng, Zhongzhong Chen, Guohui Lu, Yun Gong, Richard H. Finnell, Huasheng Xiao, Bin Qiao, Hongyan Wang

**Affiliations:** 1 Institute of Reproduction and Development at Obstetrics & Gynecology Hospital, State Key Laboratory of Genetic Engineering at School of Life Sciences, Fudan University, Shanghai, China; 2 Department of Pathology, MD Andersen Cancer Center, Houston, TX USA; 3 Dell Pediatric Research Institute, Department of Pediatrics, The University of Texas at Austin Dell Medical School, Austin, TX USA; 4 Shanghai Biotechnology Corporation, 121 Libing Road, Shanghai, China; 5 Institute of Cardiovascular Disease, General Hospital of Jinan Military Region, Jinan, China; 6 Shanghai First Maternity and Infant Hospital, Tongji University School of Medicine, Shanghai, China

**Keywords:** Cancer metabolism, Metabolic disorders

## Abstract

Primary malignant cardiac tumors (PMCTs) are extremely rare. The apparent immunity of the heart to invasive cancer has attracted considerable interest given the continuously rising incidence of cancer in other organs. This study aims to determine the conditions that could result in cardiac carcinoma and expand our understanding of cardiac tumor occurrence. We report two cases: a male (Patient-1) with primary cardiac malignant fibrous histiocytoma (MFH) and a female (Patient-2) with primary cardiac angiosarcoma. Merged genome-wide analyses of aCGH, Exome sequencing, and RNA-sequencing were performed on Patient-1 using peripheral blood, carcinoma tissue, and samples of adjacent normal tissue. Only whole-transcriptome analysis was carried out on Patient-2, due to insufficient quantities of sample from Patient-2. We identified a novel inherited loss of functional mutation of *FH* (Glu404Asp), a recurrent somatic hotspot mutation of *PIK3CA* (His1047Arg) and a somatic duplication in copy number of *HIF1A*. *FH* (E404D) severely compromised FH enzyme activity and lead to decreased protein expression in cardiac tumor tissues. We previously reported a functional mutation *ACOX2* (R409H), which is potentially associated with decreased β-oxidation of fatty acids in the cardiac tumor tissue. Results of transcriptome analyses on two patients further revealed that the RNA expression of genes in the TCA cycle and beta-oxidation were uniformly downregulated. In this study, combined effects of *FH* (E404D) and *ACOX2* (R409H) on metabolic switch from fatty acids to glucose were remarkably distinct, which might be an essential precondition to trigger the occurrence of PMCTs and mimic the Warburg effect, a hallmark of cancer metabolism.

## Introduction

Cardiac tumors are extremely rare neoplasms. The incidence of primary cardiac tumors (PCTs) is at least 100 times less common than that of metastatic cardiac tumors, with an autopsy frequency of 0.0017–0.02%^1,2^. Most PCTs are benign, and <10% of them exhibit features of malignancy. According to a recent epidemiologic study based on data generated from >7 million cancer cases, primary malignant cardiac tumors (PMCTs) account for only 0.008% of all cancer cases^3^. Approximately 65% of PMCTs are sarcomas, including angiosarcomas, undifferentiated sarcoma, malignant fibrous histiocytoma (MFH), leiomyosarcomas, and rhabdomyosarcomas, while the other 35% are lymphomas (27%) and mesotheliomas^3^. Despite dramatic advances in understanding the pathogenesis and developing more efficacious cancer therapy benefited from whole-genome analyses and targeted treatment, the incidence of cancer inevitably increases in most populations studied. Investigations attempting to better understand just how cardiac tissue resists tumor formation, and what triggers heart tumors when they do occur, will greatly increase our ability to understand the underlying mechanism of oncogenesis. In this study, we performed integrated genomic analyses including array comparative genomic hybridization (aCGH), Exome, and RNA sequencing on peripheral blood, adjacent normal tissue, and tumor tissue of a patient with cardiac MFH in the right ventricle (RV).

MFH is also referred to as an undifferentiated high-grade pleomorphic sarcoma and represents <5% of all primary cardiac malignant sarcomas^3^. There are no gender biases in the incidence of MFHs and the mean age at presentation is 47.1^2^. In 2001, Okamoto reviewed 46 cases of MFH in the literature and found that 81% of cases were located in the left atrium, while only 2 cases occurred in the RV^4^. So far, <100 cases of primary cardiac MFH have been reported worldwide. Primary cardiac angiosarcomas, although very rare, are the most common cardiac sarcomas, comprising 43% of all sarcomas. The prevalence of angiosarcomas is 2–3 times higher in men than in women. Up to 2/3 of angiosarcomas were located in the right atrium, in contrast to cardiac MFH. Previous studies have claimed that point mutations in the *POT1* (protection of telomeres 1) and *PLCG1* (phospholipase C gamma 1), which are known to be involved in telomere maintenance and apoptosis resistance, respectively, were responsible for cardiac angiosarcoma^5,6^.

Given the fundamental principle that cancers must arise from a cell with the potential to divide, two major hypotheses regarding stem cell and de-differentiation theories have been used to explain the origin of cancer^7–9^. Cardiac muscle cells are terminally differentiated; thus it is highly unlikely that tumors arise from cardiac muscle cells. However, cardiac interstitial cells, which populate the space between the cardiac muscle cells, retain the potential for self-renewal with limited differentiation. Such characteristics make these cells likely candidates for being the potential source for tumor development in the heart.

The enhanced dependence on glucose and glycolytic metabolism is a near-universal feature of advanced cancer cells, which has been called the Warburg effect^10,11^. However, nearly 95% of ATP production in the heart is derived from mitochondrial oxidative phosphorylation, whereas glycolysis accounts for only 5%^12^. Indeed, unlike most organs, heart tissues utilize fatty acids instead of glucose as a primary energy source for optimal function, which could be considered an advantage in the prevention of cardiac tumors. During the progression of heart failure, the capacity of the heart to utilize fatty acids as its primary source of energy is diminished^13^. This study aims to expand our recognition and understanding on tumor occurrence of PMCT.

## Materials and Methods

### Patient-1: a male case with primary cardiac (MFH)

**Fig. 1:**
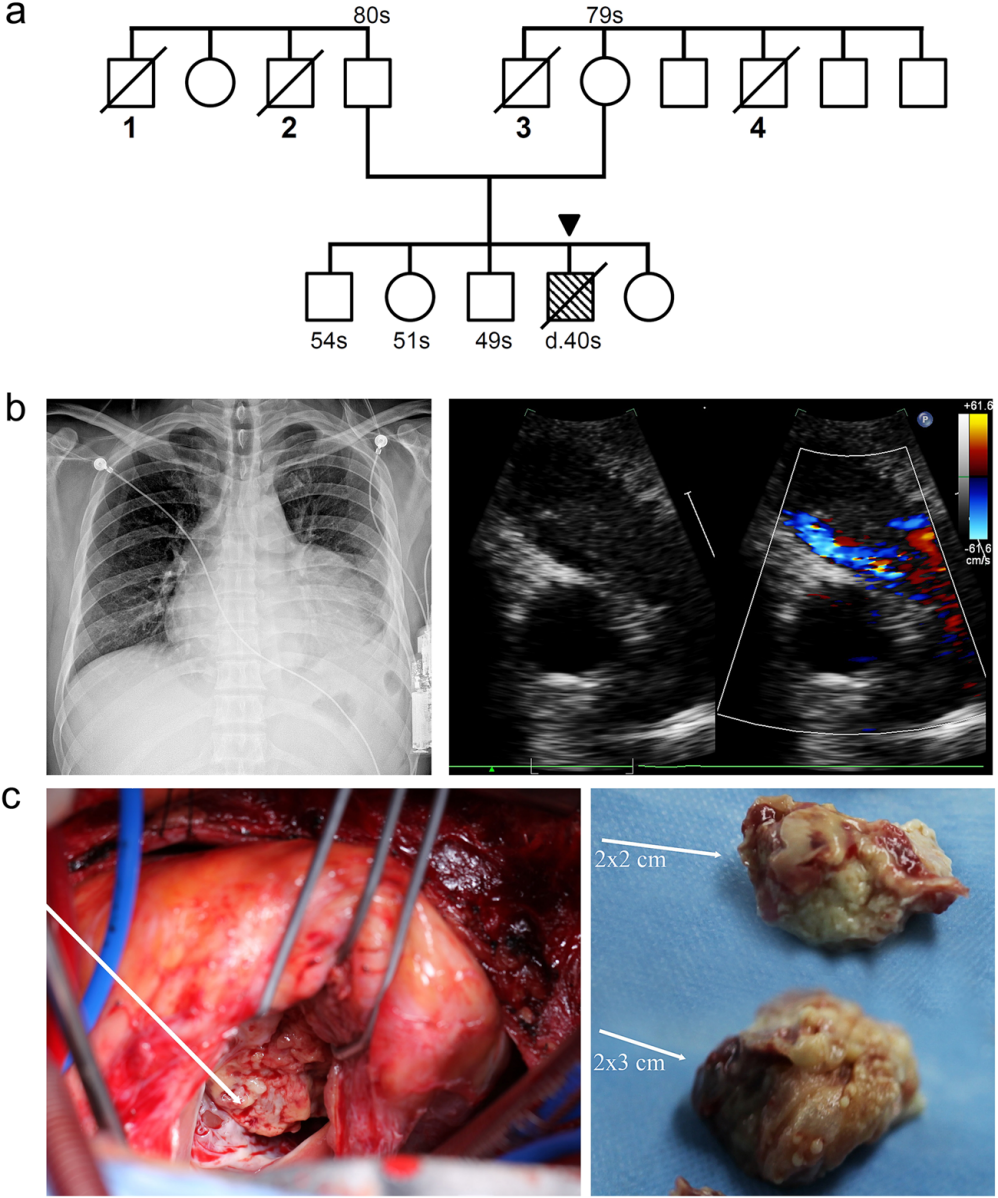
A 40-year-old male was diagnosed as having cardiac MFH (Patient-1). **a **The pedigree of the Patient-1's family. (1) Unknown reason death; (2) esophageal carcinoma; (3) neuroglioma; (4) acute myeloid leukemia. A sibling (54s) of the patient, who only carries the *FH* (E404D), once accepted the benign sarcoma resection on the back. In this study, *FH* (E404D) and *ACOX2* (R409H) were paternally and maternally derived, respectively. **b** Imaging modalities for identification and characterization of a primary cardiac tumor in Patient-1. Transthoracic echocardiogram and chest X-ray revealed increased cardio-thoracic ratio due to a 10 × 5.5 cm^2^ tumor in the right ventricle (RV). **c** The irregular and heterogenous pieces of sarcoma were resected from RV during the surgery

In November 2011, a 40-year-old man presented with sudden chest pain and shortness of breath after slight exertion. The patient had a 20-year history of smoking cigarettes and drinking alcohol. In 2003, he had undergone an operation and medicinal treatment as a result of tuberculous pleurisy. The patient had no drug/food allergy history and no history of heredity diseases in his family. However, three siblings of Patient-1's parents were diagnosed with malignant tumors and another sibling died of an unknown cause (Fig. 1a). Chest X-rays showed an enlarged cardiac shadow and abnormal cardio-thoracic proportions (Fig. 1b). Electrocardiogram detected a right bundle branch block and ST-T abnormalities. Transthoracic echocardiogram revealed pericardial effusion, space-occupying lesions, and an obstruction in the RV outflow tract with a round mass 10 × 5.5 cm^2^ in size (Fig. 1c). A full-body computed tomographic and abdominal ultrasound examination excluded tumors in other organs. Although the patient received a complete surgical resection of the abnormal tissue, he died secondary to a recurrence of the tumor 2 months post-surgery.

**Fig. 2:**
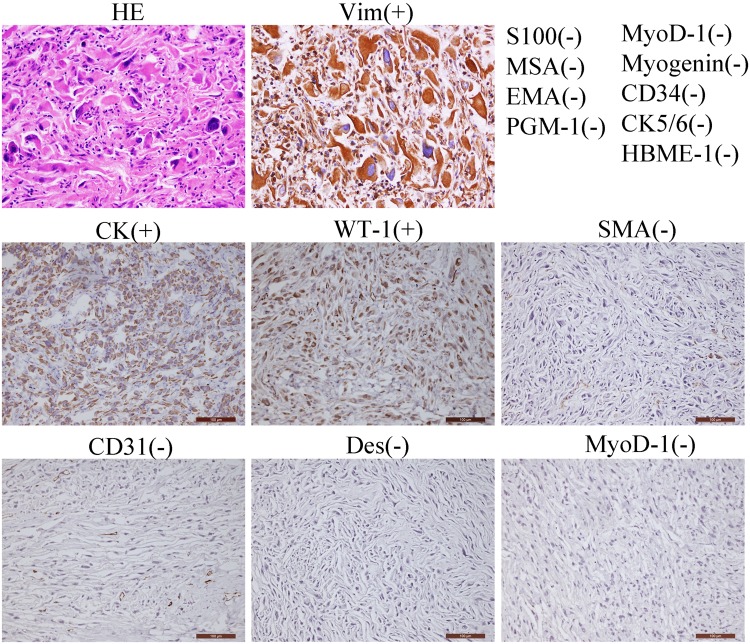
Pathological diagnosis of primary cardiac malignant fibrous histiocytoma (MFH). Light microscopy (hematoxylin–eosin, HE) presenting the morphology of tumor cells. Immunohistochemical staining positive for Vim (vimentin), CK (cytokeratin), and WT-1 (Wilms' tumor gene 1) and negative for MyoD-1 (myogenic differentiation antigen 1), CD31 (platelet endothelial cell adhesion molecule-1, PECAM-1/CD31), Desmin, S100 (calcium-binding protein), SMA (smooth muscle actin), MSA (muscle-specific Actin), EMA (epithelial membrane antigen), Myogenin, CK5/6 (cytokeratin 5/6), CD34 (hematopoietic progenitor cell antigen CD34), HBME-1 (human mesothelial cell), and PGM-1 (phosphoglucomtase-1). Representative images of the antibody staining are displayed. Scale bars, 100 µm

Peripheral blood samples were collected for heparin anticoagulation prior to surgery. Tumor and adjacent tissue samples were taken during surgery and subsequently sent to the following medical facilities for pathologic evaluation: the Shanghai No.6 People’s Hospital, Shanghai, China; the Huashan Hospital affiliated with Fudan University, Shanghai, China; and the MD Anderson Cancer Center, Houston, TX, USA. Immunohistochemical analysis was performed following the standard manufacturer’s protocols. The diagnostic report included a positive reaction for Vimentin(+), CK(+), and WT-1(+) and negative reaction for MyoD-1(−), CD31(−), CD34(−), Desmin(−), SMA(−), S100(−), MSA(−), EMA(−), Myogenin(−), CK5/6(−), HBME-1(−), and PGM-1(−) (Fig. 2). Hematoxylin and eosin staining indicated that the cells contain many multinuclear cells and abnormal nuclei. Finally, this patient was diagnosed as having a primary cardiac MFH in the RV.

### Patient-2: a female case with primary cardiac angiosarcoma

To extend the primary insights obtained from Patient-1, we obtained and analyzed whole-transcriptome data on Patient-2 via collaboration. Patient-2, a female, was diagnosed as having primary cardiac angiosarcomas at the age of 44 years in July 2012 by the Department of Pathology at Zhongshan Hospital affiliated with Fudan University, Shanghai, China. During a surgical operation, a 7 × 5.5 × 3 cm^3^ taupe-colored soft tumor was resected from the right atrium, where the tumors had already infiltrated the myocardial tissue. The diagnostic report displayed positive reaction for Calponin(+), Vimentin(+), CD34(+), CD31(+), CD68(+), SMA(+), Ki67(+), and Nestin(+) and negative reaction for MyoD-1(−), Myogenin(−), Bcl-2(−), Desmin(−), CK(−), S100(−), EMA(−), CEA(−), and F8(−) (data not shown). The patient died as a result of tumor recurrence within 1 year after the original surgical resection. All the procedures in this study were approved by the Medical Ethics Committee of Fudan University and written consent was obtained prior to commencement.

### Array comparative genomic hybridization

We have utilized three chips of aCGH analyses on Patient-1 including Chip 1 (blood/ctrl), Chip2 (tumor tissue/ctrl), and Chip 3 (tumor tissue/adjacent tissue). Agilent SurePrint G3 Human Genome CGH Microarrays were used in microarray analyses (Agilent Technologies, Santa Clara, CA, USA). Human male genomic DNA (P/N G1471; Promega Corporation, Madison, WI, USA) was used as a control for reference labeling (Ctrl). Data were extracted using the Agilent Feature Extraction software (v10.7.3.1) and analyzed with Genomic Work Bench (v7.0.4) (Agilent Technologies). Subsequently, a statistical sensitivity threshold set at 6.0 was determined for the Aberration Detection Method 2 algorithm. Our threshold settings to make a positive call were 6.0 for sensitivity, 0.4 for the minimum absolute average log2 ratio, and 3 for the minimum number of probes per region. In this study, we did not detect any germline copy number variations (CNVs) that contain cancer predisposing genes. Seven somatic CNVs including 42 genes were identified in Patient-1.

### Exome sequencing

Genomic DNA isolated from adjacent normal tissue, tumor tissue, and blood samples of Patient-1 were used to perform Exome sequencing by utilizing SureSelect XT Target Enrichment System (Agilent Technologies). Exome-enriched genomes were multiplexed by flow cell for paired-end 2 × 100 bp read sequencing, based on the protocols established for the HiSeq 2000 platform (Illumina, USA). The Genome Analysis Toolkit software package was used for single-nucleotide variant and indel (insertion and deletion) detection. On average, the efficiency of capture exceeded 92% and we identified a total of 34,987 variants from 3 listed samples.

In this study, we focused on protein-altering variants (missense, nonsense, splicing site variants, and coding indels). For germline variants, we employed an exclusion of minor allele frequency >0.1% variants in 1000 Genomes owing to the fact that causative variants of PMCTs are rare and unlikely to be catalogued in the existing 1000 Genomes or any such databases. Functional predictions were assessed with Polyphen-2 and SIFT. In this study, 86 germline and 12 somatic variants could meet our filter criteria.

### RNA-seq

RNA-sequencing was performed at the Shanghai Biotechnology Company. cDNA library was built according to the standard manufacturer’s protocol. Next, paired-end 2 × 100 bp read sequencing was performed using the Hiseq 2000 platform (IIIumina). The FASTX-Toolkit (v0.0.13) was used to trim low-quality bases. High-quality reads were aligned to the NCBI human reference genome (h19) using spliced mapping alignment in TopHat (v1.3.2). Mapped reads were used to calculate gene expression levels by Avadis NGS. Genes with coverage that exceeded 5 reads coverage were classified as positive. Gene expression level was calculated by RPKM (Reads Per Kilobase of exon model per Million mapped reads) according to the provided standard formula. Genes with expression fold-change <2 were excluded from subsequent analyses.

### Polymerase chain reaction (PCR) and Sanger sequencing

The potential pathogenic variants from Exome sequencing FH Glu404Asp (E404D) was confirmed by Sanger sequencing with primers F:5′-CTCAGGATGCTGTTCCACTTACT-3′ and R:5′-CTCTGCTGTGAGATAGCCAAGTT-3′. PCRs were amplified by using Taq^TM^ HotStart DNA Polymerase (TaKaRa Bio Inc., Shiga, Japan). PCR products were purified by Exon/SAP and sequenced by the BigDye Cycle Sequencing Kit (Thermo Fisher Scientific, Waltham, MA, USA) and ABI 3730 Genetic Analyzer (Applied Biosystems, Foster City, CA, USA).

### FH site-directed mutagenesis

The *FH* (fumarate hydratase) expression vector (pCMV6-C-Myc) was obtained from Origene (Rockville, MD, USA). The QuickChange Site-Directed Mutagenesis Kit (Stratagene, Agilent) was used to generate mutant *FH* (Glu404Asp) with primers (F: 5′-GAGGCAGCAATGGACATTTTGACTTGAATGTTTTCAAGCC-3′ and R: 5′-GGCTTGAAAACATTCAAGTCAAAATGTCCATTGCTGCCTC-3′). Mutants were then confirmed by Sanger sequencing.

### FH activity measure and fumarate quantification

Fumarase Activity Colorimetric Assay Kit (K596-100; Biovision) and Fumarate Assay Kit (MAK060; Sigma) were used. Briefly, human HEK 293T cell line was transfected with wild-type and mutant E404D FH. After 36 h, 2 × 10^5^ cells were lysed in either fumarase assay buffer or fumarate assay buffer from the corresponding kit. Then developer and enzyme mix from the Kit were added and incubated at 37 °C by following standard protocol (30 min for enzyme activity assay, 60 min for fumarate assay). The absorbance intensity was read at 450 nm using a Multiskan MK3 Spectrophotometer (Thermo Fisher).

### Native Gel

After transfection, HEK 293T cells were lysed in nondenature lysis buffer (C510013; Sangon Biotech), and then anti-Myc immunoprecipitation (IP) experiment was performed by standard procedures. Myc peptides were used to elute the FH recombinant protein from beads. Supernatants were collected and added with 5× protein nondenaturing loading buffer (C506032; Sangon Biotech). 10× Tris-Glycine Gel Running Buffer (Nondenature Buffer) (C506032, Sangon Biotech) was diluted to prepare working solution. All the above steps were performed on ice or 4 °C. Native gels were finally stained by the Rapid Protein Silver Kit (C500029; Sangon Biotech).

### Immunoprecipitation

HEK 293T cells were transfected with wild-type and mutant FH by Lipofectamine 3000 (Thermo Fisher). After 36 h, cells were lysed in Pierce IP Lysis Buffer (87787, Thermo Fisher) supplemented with or without work concentration of protein crosslinker (S1885, disuccinimidyl suberate, Sigma) depending on different conditions. After centrifugation, 200 µg lysates were incubated with anti-His antibody to proceed with the standard IP experiment.

### Immunoblot analysis

Cardiac tumor and adjacent normal tissues were homogenized in RIPA Buffer (Sangon Biotech) supplemented with a 1:200 dilution of protein inhibitor (Sangon Biotech). Following electrophoresis and membrane transfer, proteins were probed with one of the following primary antibodies in either 5% non-fat milk or 5% bovine serum albumin: anti-FH (#4567), anti-G6PD (# 12263), and anti-HIF-1A (# 3716) from CST (Cell Signaling Technology); as well as anti-PFKM (Abcam, Cambridge, UK), anti-β-Actin (A1978; Sigma-Aldrich), anti-GAPDH (G9545; Sigma-Aldrich), and anti-Myc (TA150121; Origene). After incubation with secondary horseradish peroxidase antibodies (either anti-rabbit [7074; CST] or anti-mouse [8270; Sigma-Aldrich]), chemiluminescent detection was performed using ECL1/2 (PC198506/1859701; Thermo Fisher).

### Statistics

All data are presented as individual samples and mean ± SEM. Student’s two-tailed unpaired *t* tests were used to determine statistical significance of differences between FH wild type and mutant using the GraphPad Prism software v. 5.01 (GraphPad Software, Inc., La Jolla, CA, USA). Differences were considered to be significant when *P* values were <0.05.

## Results

### Inherited loss-of-function mutation of *FH* (E404D) was identified in Patient-1

**Fig. 3:**
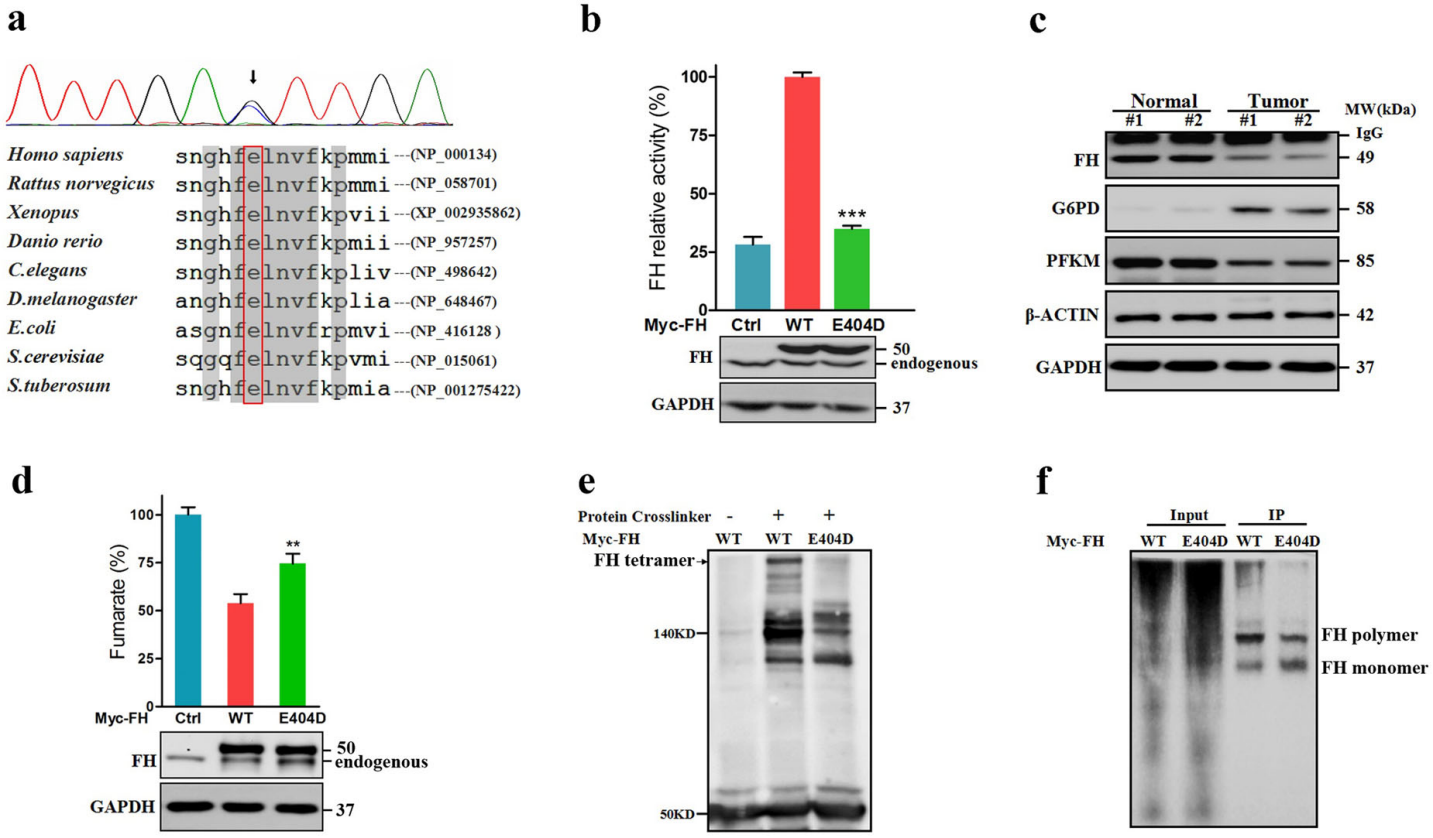
*FH* (E404D) was identified in Patient-1 by exome sequencing. **a** Sanger sequencing and sequencing alignment of *FH* (E404D) (NCBI reference sequences are shown on the right). Black arrow indicated that the heterozygous mutation in FH (c.1212G>C, p.Glu404Asp) was further confirmed by Sanger sequencing. *C.elegans* *Caenorhabditis elegans*, *D.melanogaster* *Drosophila melanogaster*, *E.coli* *Escherichia coli*, *S.cerevisiae* *Saccharomyces cerevisiae*, *S.tuberosum* *Solanum tuberosum*. **b** FH Glu404Asp significantly compromised enzyme activity by overexpressing recombinant FH-Myc protein in HEK 293T cells in vitro (***P < 0.001). An empty vector served as a control (Ctrl). Relative activity is presented as the mean ± SE of four independent experiments; each sample was assayed in quadruplicate in each experiment. **c** Expression of FH was downregulated, whereas G6PD (glucose-6-phosphate dehydrogenase) were upregulated in the cardiac tumor tissue when compared with adjacent normal tissue. GAPDH and β-ACTIN served as loading controls. Nt adjacent normal tissue, Tt tumor tissue. **d** FH E404D significantly increased levels of cellular fumarates in vitro (**P < 0.01). HEK 293T cells were transfected and an empty vector served as a control. **e** FH (E40ED) affected normal tetramer formation. HEK 293T cells were transfected with Myc-tagged wild type or (E40D) FH for recombinant overexpression. A 1:100 dilution of a protein crosslinker (PC) was added to the RIPA buffer before cell lysis. Subsequently, anti-Myc immunoprecipitation and anti-FH immunoblotting were performed following the standard procedures. A 50-KDa band was indicative of FH-Myc recombinant protein. After PC treatment, a 220-KDa band (black arrow) was generated, corresponding to an FH tetramer. This band was significantly decreased in cells transfected with mutant FH. **f** Native gels assay was performed to test the polymer formation of FH. After anti-Myc immunoprecipitation, two different bands were generated. The higher band, corresponding to polymer, was decreased in the cells transfected with mutant FH

Using exome sequencing, we identified 86 germline and 12 somatic variants that could meet our filter criteria (Supplementary file). According to Catalogue of Somatic Mutations in Cancer (COSMIC) database and 60 autosomal-dominant genes that were classified by Downing and colleagues^14,15^, we identified a paternal-derived germline mutation p.Glu404Asp(E404D) in FH (Fig. 3a). FH is an enzyme that converts fumarate to malate in the tricarboxylic acid cycle (TCA) cycle. In this study, E404D is a novel variant in dbSNP132, 1000 Genomes, and ExAC database (0/121296) and was located in the protein-binding site for tetramer formation, which is essential for enzyme activity. E404D was uniformly predicted to damage protein function by six different software packages, including SIFT, Poly-phen2, Provean, Mutationtaster, Mutationassessor, and Fathmn (Table 1). This amino acid substitution was extremely conserved across taxa and even some bacteria (*Escherichia coli and Saccharomyces cerevisiae*) and plants (*Solanum tuberosum*). Assays of enzymatic activity indicated that FH (Glu404Asp) significantly compromised FH activity by 65.08 ± 2.367% (*P* < 0.001) in vitro compared with overexpressed wild-type recombinant proteins (Fig. 3b). Western blotting indicated that protein expression of *FH* was markedly depleted in tumor tissues (Fig. 3c). As a consequence, cellular fumarate levels were increased by 20.29 ± 6.343% (Fig. 3d) as a result of the inability of FH to form tetramers in HEK 293T cells (Fig. 3e, f).

**Table 1:** Functional predictions of FH p.Glu404Asp by six different software packages

Gene name	FH
Amino acid change	Glu404Asp
MAF in 1000 Genome	0.00
MAF in ExAC	0.00
Protein-binding sites	Tetramer formation
SIFT	0.001 Damaging
Polyphen-2	0.958 Probably damaging
Provean	−2.94 Deleterious
Mutationassessor	2.925 Medium
Mutationtaster	Disease causing
Fathmm (inherited disease)	−6.29 Damaging

*MAF* minor allele frequency

### Duplication in HIF1A is correlated with enhanced HIF1A expression in Patient-1

**Fig. 4:**
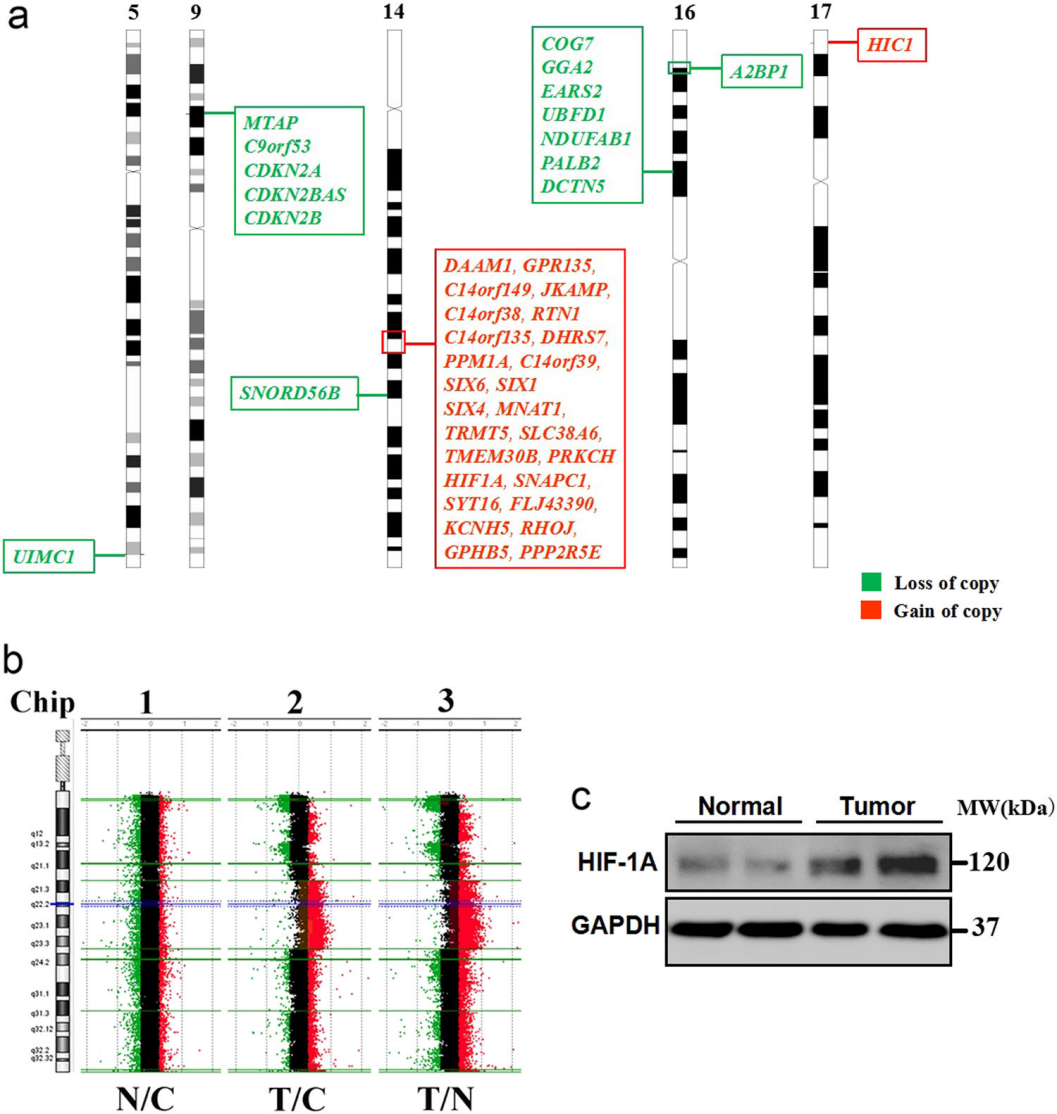
Somatic HIF1A duplication was identified in cardiac tumors of Patient-1 by aCGH **a** Seven somatic CNVs including 42 genes were identified by aCGH (14q23.1–23.2,17p13.2, 5q35.2, 9p21.3, 14q24.2, 14p12, 16p12.2, and 16p13.3). **b** A larger fragment of somatic duplication including *HIF1A* on locus 14q23.1-23.2 was identified in both Chip-2 and Chip-3. N adjacent normal tissue, C control for reference labeling, T tumor tissue. **c** HIF1A expression in cardiac tumor tissues was markedly upregulated. GAPDH served as a loading control. Representative image from three independent experiments was displayed

Using aCGH micro-arrays, 6 germline and 7 cancer-specific (somatic) CNVs with |log2ratio| **>** 0.4 were identified (Supplementary file). All 6 germline CNVs resulted from loss of copy numbers, which contains three genes including *RGS7*, *MFSD1*, and *MARCH1*. However, no studies have indicated that these three genes are associated with the occurrence of caner to this day. In this study, 7 identified somatic CNVs contains a total of 42 genes (Fig. 4a). To determine the causative CNVs, we utilized the latest Cancer Gene Census (CGS) in the COSMIC, which contain 595 cancer predisposing genes. Three candidate genes including a duplication in *HIF1ɑ* (hypoxia-inducible factor 1-alpha), and deletions in autosomal dominant genes *CDKN2A* (cyclin-dependent kinase inhibitor 2A) and *PALB2* (partner and localizer of *BRCA2*) were identified from candidate gene list of CGS (Fig. 4b). Subsequent RNA-seq and western blot revealed that the expression of HIF1ɑ was closely correlated with HIF1A duplication in tumor tissues, whereas gene expression of CDKN2A and PALB2 was not significantly changed (fold-change < 2) (Fig. 4c).

### Recurrent hotspot somatic mutation in PIK3CA (H1047R) was first identified in PCT

**Fig. 5:**
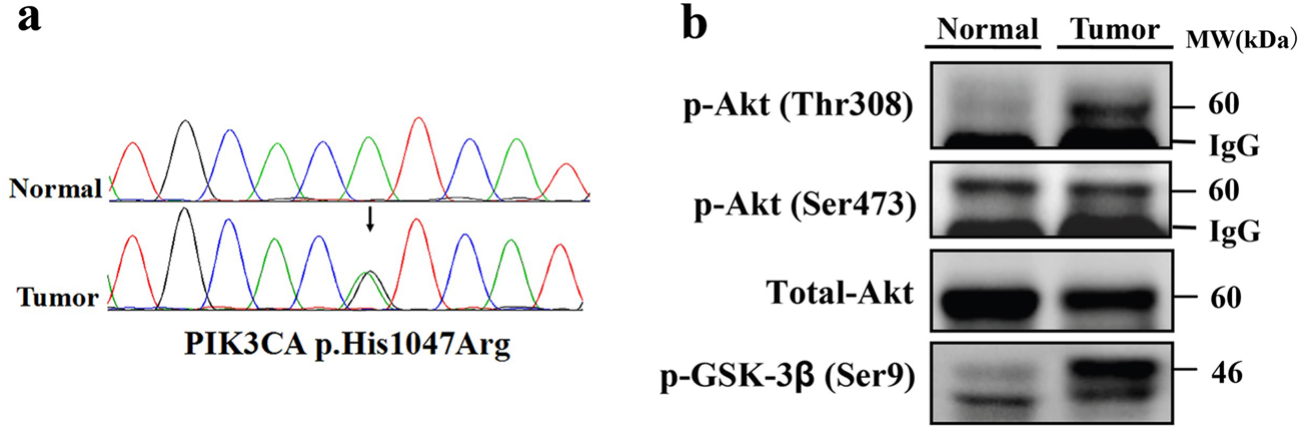
Recurrent hotspot PI3KCA (H1047) was first identified in cardiac tumor tissue. **a** Sanger sequencing of PIK3CA (His1047Arg) by using blood and tumor tissue samples of Patient-1. **b** Phosphorylation of Akt Thr 308 site as well as GSK3-beta at Ser9 site was markedly enhanced in cardiac tumor tissues

PI3KCA H1047R is a recurrent hotspot mutation in human cancers, especially in breast cancers. Although somatic mutations in PIK3CA have been reported in many human cancers^16,17^, in this study we first identified PIK3CA (H1047) in cardiac tissues (Fig. 5a). To investigate the effect of PIK3CA (H1047R) on activation of protein kinase B/Akt signaling pathway, we tested the phosphorylation of pathway members by western blotting in cardiac tumor tissues. The results showed phosphorylation of AKT at the Thr308 site and GSK-3β at Ser9 site was markedly increased compared to the expression in adjacent normal tissues (Fig. 5b). PIK3CA (H1047R) was reported to induce and reactivate multipotency and multi-lineage mammary tumors^18,19^.

### Metabolic switch from fatty acid to glucose in both Patient-1 and -2

RNA-seq revealed 6092 differentially expressed genes (with >2× fold-change in Patient-1. To determine the functional relevance of these differentially expressed genes, we utilized an ingenuity pathway analysis approach to identify the top enriched pathways. Notably, the expression of all genes in mitochondrial and peroxisomal beta-oxidation of fatty acids and TCA cycle were entirely downregulated (Table 2). Meanwhile, the glycolytic pathway was mostly switched over to the pentose phosphate pathway (PPP) (Table 3). To further validate our conclusions regarding the metabolic switch, we analyzed the results of the transcriptomic data from Patient-2. We found that the patterns of metabolic switch observed in this second case were highly consistent with that observed in Patient-1 (Tables 2 and 3). Moreover, the depleted FH and elevated HIF1A expression were also observed in Patient-2.

**Table 2:** Gene expression of the TCA cycle genes was entirely downregulated in tumor tissues of both Patient-1 and -2 via whole-transcriptome analysis

Gene name		Fold-change (tumor/normal)	
		Patient-1	Patient-2
CS	Citrate synthase	0.28	0.45
ACO1	Aconitase 1, soluble	NA	0.77
ACO2	Aconitase 2, mitochondrial	0.031	0.17
IDH1	Isocitrate dehydrogenase 1 (NADP+), soluble	3.27	2.11
IDH2	Isocitrate dehydrogenase 2 (NADP+), mitochondrial	0.21	0.52
IDH3A	Isocitrate dehydrogenase 3 (NAD+) alpha	0.13	0.28
IDH3B	Isocitrate dehydrogenase 3 (NAD+) beta	0.26	0.49
IDH3G	Isocitrate dehydrogenase 3 (NAD+) gamma	1.09	0.65
OGDH	Oxoglutarate (alpha-ketoglutarate) dehydrogenase	0.18	0.19
OGDHL	Oxoglutarate dehydrogenase-like	0.12	0.12
DLD	Dihydrolipoamide dehydrogenase	0.11	0.45
DLST	Dihydrolipoamide S-succinyltransferase	0.43	0.68
SUCLG1	Succinate-CoA ligase, alpha subunit	0.30	0.38
SUCLG2	Succinate-CoA ligase, GDP-forming, beta subunit	0.22	0.57
SUCLA2	Succinate-CoA ligase, ADP-forming, beta subunit	0.12	0.39
SDHA	Succinate dehydrogenase complex, subunit A,	0.10	0.46
SDHB	Succinate dehydrogenase complex, subunit B,	0.19	0.33
SDHC	Succinate dehydrogenase complex, subunit C,	0.39	0.63
SDHD	Succinate dehydrogenase complex, subunit D,	NA	0.63
FH	Fumarate hydratase	0.20	0.22
MDH1	Malate dehydrogenase 1, NAD (soluble)	0.091	0.20
MDH2	Malate dehydrogenase 2, NAD (mitochondrial)	0.17	2.35
GOT1	Glutamic-oxaloacetic transaminase 1, soluble	0.047	0.13
GOT2	Glutamic-oxaloacetic transaminase 2, mitochondrial	0.16	0.22

**Table 3:** Glucose metabolism was mostly switched from glycolysis to pentose phosphate pathway in both patients

Gene name		Fold-change (tumor/normal)	
		Patient-1	Patient-2
*Glycolysis⇒PPP pathway*			
HK1	Hexokinase 1	0.24	0.45
HK2	Hexokinase 2	3.38	1.67
HK3	Hexokinase 3	21.30	1.89
PFKM	Phosphofructokinase, muscle	0.12	0.16
FBP1	Fructose-1,6-bisphosphatase 1	60.13	1.43
G6PD	Glucose-6-phosphate dehydrogenase	7.13	3.20
PGLS	6-Phosphogluconolactonase	4.74	1.98
PGD	Phosphogluconate dehydrogenase	2.49	7.17

## Discussion

We previously reported a functional germline mutation, R409H, in *ACOX2* (acyl-CoA oxidase 2) in this patient, which is potentially associated with decreased β-oxidation of fatty acids^20^. In this study, *FH* (E404D) and *ACOX2* (R409H) are paternally and maternally derived, respectively. The Patient-1's family is prone to tumor occurrence where three siblings of his parents were diagnosed with malignant tumors, including esophageal carcinoma, neuroglioma, and acute myeloid leukemia, and another sibling died of an unknown cause. The accumulation of pathogenic mutations inherited from both parents greatly increased the risk of cancer in Patient-1.

Germline mutations in FH have previously been reported in FH-deficiency syndrome, multiple cutaneous and uterine leiomyomatosis, hereditary leiomyomatosis and renal cancer, and neuroblastoma^21,22^. As a autosomal-dominant cancer predisposing gene, FH loss dysregulates glucose metabolism and causes combined respiratory chain defects^23^. Increased fumarates also induce epithelial-to-mesenchymal transition in FH-deficient cells^24^. ACOX2 is an enzyme that is involved in the degradation of bile acid intermediates and branched fatty acids. Deficiency in ACOX2 leads to accumulation of C27 serum bile acids^25^. A recent study by Desai et al. found that excess bile acids suppress fatty acid oxidation and lead to metabolic switch in the heart^26^, which is mostly accordance with our previous findings. Although FH deficiencies promote a stabilization of HIF1A expression secondary to increased cellular fumarates^27^, the expression of most HIF1 downstream targets, including vascular endothelial growth factor A (VEGFA) and VEGFB, were unexpectedly decreased in tumor tissue due to a truncated HIF1ɑ splice variant lacking exon 10 in Patient-1^28^.

**Fig. 6:**
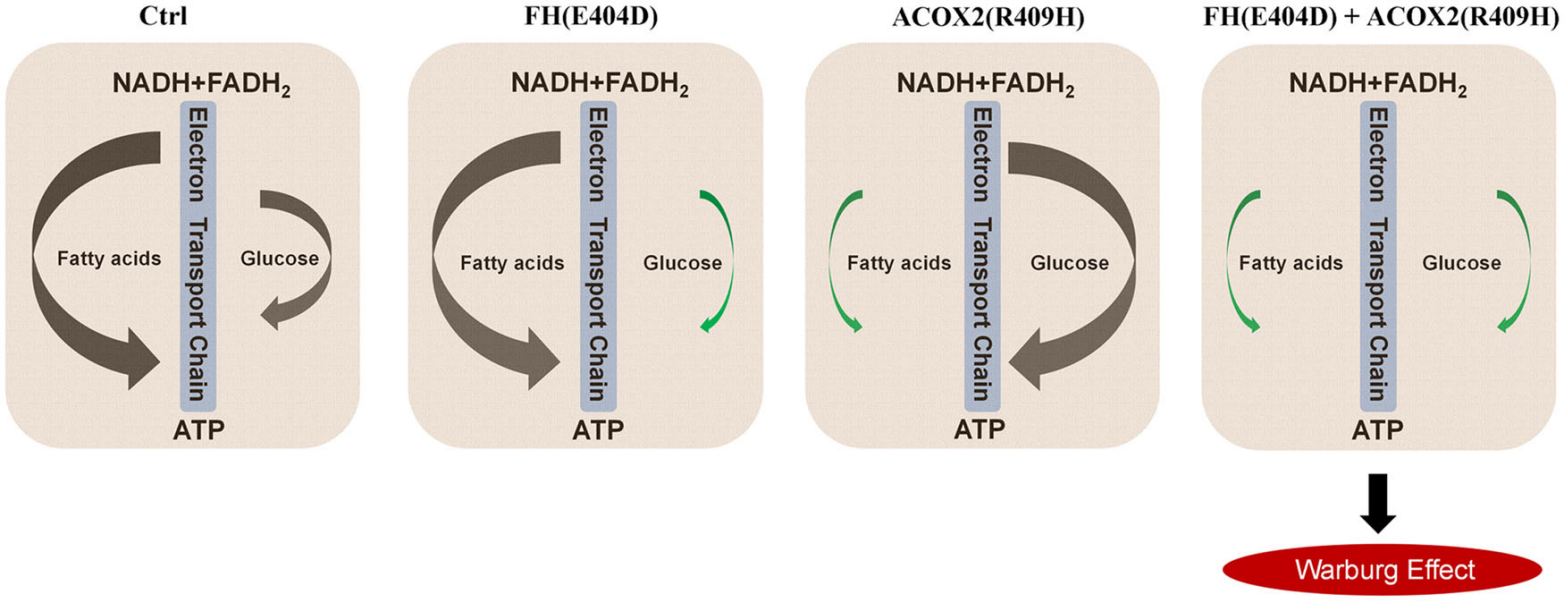
Proposed pathogenic model to explain the metabolic defects in cardiac tumor tissues of Patient-1. Under normal condition, cardiac cells prefer to utilize fatty acids as primary fuels (Left). *FH* (E404D) and *ACOX2* (R409H) could independently take effect in oxidation of fatty acids and TCA cycle as metabolic enzymes, respectively (Middle). These two mutations probably have combined effects on metabolic switch from fatty acids to glycolysis. Green arrows indicate downregulated metabolic processes according to RNA-seq data from Patient-1

If Warburg effect works widely, the fact that heart tissue utilizes fatty acids instead of glucose uptake as primary fuels may naturally facilitate its tumor resistant feature, although it is still unclear whether blocked fatty acid utilization is the necessary prerequisite for cardiac tumor initiation. Accumulated serum bile acids caused by ACOX2 depletion might decrease fatty acid oxidation. Owing to the fumarase deficiency caused by *FH* (E404D), glucose utilization in the heart was further switch from aerobic respiration to anaerobic glycolysis, which mimic the Warburg effect (Fig. 6). Although we fail to provide solid evidence to support that germline *FH* (E404D) and *ACOX2* (R409H) are a direct cause of tumorigenesis in the heart, our results suggested that these two variants have combined effects on metabolic dysfunction in the cardiac tumor.

## Electronic supplementary material

7 somatic CNV

86 germline variants

12 somatic mutations
