# Supplementary material for: Combined effects of FH (E404D) and ACOX2 (R409H) cause metabolic defects in primary cardiac malignant tumor
Source: Cell Death Discov. 2018 Jul 23;4:70. doi: 10.1038/s41420-018-0072-3 (PMC6056498; doi:10.1038/s41420-018-0072-3)
Supplement: Supplementary file 1 — 7 somatic CNV [file 41420_2018_72_MOESM1_ESM.pdf]

Table S1. 42 genes were included in the 7 somatic CNVs with absolute log2ratio value > 0.4 in both Chip-2 and Chip-3.

Cytoband	Start	End	Length/bp	log2ratio		Genes in Fragment
				Chip-2 Tt-Ctrl	Chip-3 Tt-Nt	
5q35.2	176349059	176441899	92841	-0.6	-0.6	*UIMC1*
9p21.3	21863204	22052139	188936	-0.4	-0.4	*MTAP，C9orf53，CDKN2A，CDKN2BAS，CDKN2B*
14q24.2	71826496	71951470	124975	-0.5	-0.6	*SNORD56B*
16p13.3	5441115	7072584	1631470	-0.5	-0.5	*A2BP1*
16p12.2	23442748	23671709	228962	-0.4	-0.4	*COG，GGA，EARS，UBFD1，NDUFAB1，PALB2，DCTN5*
14q23.1-23.2	59601248	63922169	4320922	0.4	0.4	*DAAM1, GPR135, C14orf149, JKAMP, C14orf38, RTN1, C14orf135, DHRS7, PPM1A, C14orf39, SIX6 , SIX1 , SIX4, MNAT1, TRMT5, SLC38A6, TMEM30B, PRKCH, HIF1A, SNAPC1, SYT16, FLJ43390, KCNH5, RHOJ, GPHB5, PPP2R5E*
17p13.2	1959569	1959686	118	1.5	1.4	*HIC1*
