# Supplementary material for: Combined effects of FH (E404D) and ACOX2 (R409H) cause metabolic defects in primary cardiac malignant tumor
Source: Cell Death Discov. 2018 Jul 23;4:70. doi: 10.1038/s41420-018-0072-3 (PMC6056498; doi:10.1038/s41420-018-0072-3)
Supplement: Supplementary file 2 — 86 germline variants [file 41420_2018_72_MOESM2_ESM.pdf]

**Table S2. 86 germline nonsynonymous mutations were identified in blood sample of patient-1 by Exome sequencing.**

| #Symbol | Location | Transcript | Protein_position | Amino_acids | Codons |
|---|---|---|---|---|---|
| ACOX2 | 3:58512313 | ENST00000459701 | 395 | R/H | cGc/cAc |
| ADAMTS14 | 10:72513635 | ENST00000373208 | 940 | L/M | Ctg/Atg |
| ADAP2 | 17:29276342 | ENST00000581548 | 110 | R/H | cGt/cAt |
| ADGRG4 | X:135430483 | ENST00000370652 | 1540 | S/P | Tct/Cct |
| AHNAK | 11:62303548 | ENST00000528508 | 8 | R/Q | cGg/cAg |
| ALCAM | 3:105264151 | ENST00000306107 | 359 | S/N | aGc/aAc |
| ALDH4A1 | 1:19209799 | ENST00000538309 | 133 | N/H | Aac/Cac |
| ANKRD36 | 2:97877478 | ENST00000461153 | 1157 | V/M | Gtg/Atg |
| ANXA9 | 1:150958836 | ENST00000368947 | 166 | D/G | gAc/gGc |
| ARPIN | 15:90446572 | ENST00000560096 | 70 | C/Y | tGt/tAt |
| ATP6V1E2 | 2:46739684 | ENST00000306448 | 56 | Y/C | tAt/tGt |
| BRCA2 | 13:32911755 | ENST00000544455 | 1088 | P/H | cCt/cAt |
| BTN3A3 | 6:26451927 | ENST00000490254 | 138 | T/M | aCg/aTg |
| CA2 | 8:86392912 | ENST00000285379 | 226 | R/H | cGt/cAt |
| CEP78 | 9:80879095 | ENST00000376598 | 496 | N/K | aaT/aaA |
| CFAP54 | 12:96900715 | ENST00000298953 | 193 | L/V | Ttg/Gtg |
| CTSH | 15:79227412 | ENST00000528741 | 29 | T/P | Acc/Ccc |
| CX3CR1 | 3:39307666 | ENST00000542107 | 112 | G/A | gGc/gCc |
| DDX53 | X:23019317 | ENST00000327968 | 381 | M/I | atG/atT |
| DHODH | 16:72048535 | ENST00000572887 | 133 | V/A | gTc/gCc |
| DNAH3 | 16:20966299 | ENST00000261383 | 3636 | P/H | cCc/cAc |
| FAN1 | 15:31218141 | ENST00000362065 | 829 | Q/H | caG/caC |
| FH | 1:241665767 | ENST00000366560 | 404 | E/D | gaG/gaC |
| GPR176 | 15:40099241 | ENST00000543580 | 86 | T/A | Acc/Gcc |
| GPRIN2 | 10:46999019 | ENST00000374317 | 47 | V/M | Gtg/Atg |
| GPRIN2 | 10:46999484 | ENST00000374317 | 202 | G/W | Ggg/Tgg |
| GPRIN2 | 10:46999922 | ENST00000374314 | 348 | V/L | Gtg/Ttg |
| GRIK1 | 21:31015249 | ENST00000399913 | 332 | A/V | gCc/gTc |
| HELZ2 | 20:62198745 | ENST00000467148 | 656 | V/M | Gtg/Atg |
| HYDIN | 16:71101200 | ENST00000288168 | 707 | T/A | Aca/Gca |
| HYDIN | 16:71098649 | ENST00000393552 | 342 | N/D | Aat/Gat |
| IMMT | 2:86400824 | ENST00000254636 | 37 | P/S | Cct/Tct |
| KIAA1683 | 19:18368720 | ENST00000600359 | 892 | R/H | cGt/cAt |
| KIAA1755 | 20:36850938 | ENST00000435901 | 76 | G/D | gGc/gAc |
| KLHL10 | 17:39998487 | ENST00000293303 | 203 | V/I | Gta/Ata |
| KRT40 | 17:39140272 | ENST00000377755 | 85 | F/S | tTc/tCc |
| LAMA4 | 6:112430669 | ENST00000230538 | 1815 | V/I | Gta/Ata |
| LAMA4 | 6:112457383 | ENST00000522006 | 1112 | P/R | cCt/cGt |
| LILRB4 | 19:55179364 | ENST00000391736 | 414 | Q/R | cAg/cGg |
| LIPH | 3:185245370 | ENST00000296252 | 177 | L/R | cTc/cGc |

| | | | | | |
|---|---|---|---|---|---|
| LRRC49 | 15:71329621 | ENST00000560369 | 608 | R/C | Cgt/Tgt |
| MAP3K9 | 14:71197492 | ENST00000553414 | 707 | R/C | Cgc/Tgc |
| MBD5 | 2:149226897 | ENST00000404807 | 462 | S/L | tCa/tTa |
| METTL21B | 12:58166617 | ENST00000333012 | 37 | G/E | gGg/gAg |
| MST1R | 3:49934990 | ENST00000344206 | 670 | V/G | gTa/gGa |
| MXRA5 | X:3238733 | ENST00000217939 | 1665 | P/S | Cca/Tca |
| NEB | 2:152390806 | ENST00000172853 | 5413 | R/W | Cgg/Tgg |
| NEDD9 | 6:11190208 | ENST00000379446 | 632 | V/I | Gtc/Atc |
| NLRX1 | 11:119052976 | ENST00000409265 | 843 | A/V | gCg/gTg |
| NOX5 | 15:69329422 | ENST00000260364 | 397 | H/Y | Cat/Tat |
| NT5C3B | 17:39983808 | ENST00000415460 | 183 | S/C | tCt/tGt |
| OR4M2 | 15:22368862 | ENST00000332663 | 96 | G/E | gGa/gAa |
| OSCAR | 19:54598604 | ENST00000358375 | 225 | Y/S | tAc/tCc |
| PARD3 | 10:34637007 | ENST00000346874 | 702 | P/S | Ccc/Tcc |
| PDE4DIP | 1:144866643 | ENST00000530130 | 24 | R/C | Cgc/Tgc |
| PDE4DIP | 1:144994658 | ENST00000369351 | 25 | R/L | cGc/cTc |
| PHKB | 16:47536987 | ENST00000455779 | 124 | R/C | Cgt/Tgt |
| PHYH | 10:13330389 | ENST00000263038 | 217 | L/V | Ctg/Gtg |
| PSMD10 | X:107328208 | ENST00000217958 | 226 | G/D | gGt/gAt |
| PTPRT | 20:40944422 | ENST00000373187 | 694 | P/A | Cct/Gct |
| PZP | 12:9312951 | ENST00000261336 | 1003 | T/M | aCg/aTg |
| RGPD3 | 2:107073501 | ENST00000409886 | 111 | D/N | Gat/Aat |
| SEC31B | 10:102257953 | ENST00000370345 | 566 | L/F | Ctc/Ttc |
| SLC25A47 | 14:100792549 | ENST00000361529 | 43 | T/M | aCg/aTg |
| SPATA6 | 1:48764419 | ENST00000371847 | 478 | C/Y | tGt/tAt |
| SPTBN2 | 11:66483347 | ENST00000529997 | 88 | G/E | gGa/gAa |
| SRRT | 7:100482950 | ENST00000388793 | 425 | R/C | Cgc/Tgc |
| SSX5 | X:48054740 | ENST00000347757 | 19 | E/Q | Gag/Cag |
| TAS1R2 | 1:19166157 | ENST00000375371 | 819 | P/L | cCg/cTg |
| TEAD2 | 19:49852034 | ENST00000539846 | 93 | R/W | Cgg/Tgg |
| TMEM70 | 8:74888679 | ENST00000517439 | 55 | G/W | Ggg/Tgg |
| TMPRSS5 | 11:113565252 | ENST00000299882 | 245 | G/S | Ggc/Agc |
| TTC28 | 22:28394576 | ENST00000397906 | 1691 | K/E | Aag/Gag |
| TTC38 | 22:46674494 | ENST00000422713 | 135 | G/A | gGc/gCc |
| TTLL12 | 22:43568512 | ENST00000216129 | 464 | V/M | Gtg/Atg |
| TUBGCP6 | 22:50657782 | ENST00000248846 | 1473 | Q/H | caG/caT |
| UTP20 | 12:101736761 | ENST00000261637 | 1447 | D/N | Gac/Aac |
| VDR | 12:48272895 | ENST00000395324 | 1 | M/T | aTg/aCg |
| VPS39 | 15:42454577 | ENST00000348544 | 771 | A/T | Gct/Act |
| WDR19 | 4:39241899 | ENST00000399820 | 789 | G/D | gGt/gAt |
| WDR34 | 9:131397002 | ENST00000372715 | 394 | P/S | Ccc/Tcc |
| WDR47 | 1:109547332 | ENST00000357672 | 351 | T/I | aCa/aTa |
| ZNF100 | 19:21909948 | ENST00000358296 | 389 | T/I | aCt/aTt |
| ZNF66 | 19:20989989 | ENST00000344519 | 528 | K/M | aAg/aTg |

| | | | | | |
|---|---|---|---|---|---|
| ZNF776 | 19:58265373 | ENST00000317178 | 292 | Y/C | tAt/tGt |
| ZNF90 | 19:20229486 | ENST00000418063 | 375 | G/S | Ggc/Agc |
