# Supplementary material for: Combined effects of FH (E404D) and ACOX2 (R409H) cause metabolic defects in primary cardiac malignant tumor
Source: Cell Death Discov. 2018 Jul 23;4:70. doi: 10.1038/s41420-018-0072-3 (PMC6056498; doi:10.1038/s41420-018-0072-3)
Supplement: Supplementary file 3 — 12 somatic mutations [file 41420_2018_72_MOESM3_ESM.pdf]

Table S3. 12 somatic nonsynonymous mutations (cancer-specific) were identified in cardiac tumor tissues.

Gene	Full names	Location	Tt	Nt	AA variant
TPR	*translocated promoter region, nuclear basket protein*	chr1:186325455	T/G	T	L617F
POMC	*proopiomelanocortin*	chr2:25384276	A/G	A	Y160H
PIK3CA	*phosphatidylinositide-3-kinase, catalytic, subunit alpha*	chr3:178952085	A/G	A	H1047R
TMEM8C	*transmembrane protein 8C*	chr9:136380644	G/A	G	A162V
KIF21A	*kinesin family member 21A*	chr12:39701378	C/T	C	W1477*
PFKM	*phosphofructokinase, muscle*	chr12:48528736	G/A	G	R253H
KIF23	*kinesin family member 23*	chr15:69732348	A/G	A	K607E
MYH3	*myosin, heavy chain 3, skeletal muscle, embryonic*	chr17:10533774	G/T	G	A1763D
JUP	*junction plakoglobin*	chr17:39919518	A/C	A	V405G
PIEZO2	*piezo type mechanosensitive ion channel component 2*	chr18:10855358	A/C	A	Y304D
SETBP1	*SET binding protein 1*	chr18:42532328	G/A	G	R1008H
ROBO3	*roundabout guidance receptor 3*	chr11:124735480	C/A	C	R3S
